# A case report on digital preoperative design, clinical application and finite element analysis for a patient with ankylosing spondylitis kyphosis

**DOI:** 10.3389/fbioe.2023.1220102

**Published:** 2023-09-12

**Authors:** Lei Zhu, Chi Zhang, Li Peng, Zifei Cheng, Xiuwen Liang

**Affiliations:** ^1^ Department of Orthopedics, Hulunbeir People’s Hospital, Hulunbuir, China; ^2^ Department of Endocrinology, Hulunbeir People’s Hospital, Hulunbuir, China; ^3^ Department of Science and Education Section, Hulunbeir People’s Hospital, Hulunbuir, China; ^4^ Department of Cardiology, Hulunbuir Zhong Meng Hospital, Hulunbuir, China

**Keywords:** ankylosing spondylitis, protrusion deformity surgery simulation, finite element analysis, digital model, AS-related kyphosis

## Abstract

**Objective:** By assessing a case of ankylosing spondylitis (AS) after thoracic lumbar protrusion deformity in a digital model and verifying its effectiveness after surgery for orthopaedic surgery process simulation, a finite element model was established for biomechanical experiments.

**Method:** A 56-year-old patient with AS underwent preoperative thoracic lumbar spine computed tomography. The data were reconstructed using MIMICS16.0 software and modelled to design and measure the nailing parameters. A three-dimensional model was established using ANSYS14.0 software, and the actual surgical procedure was simulated using biomechanical experiments. The model was verified by comparing the X-ray films obtained from patients during preoperative forward bending, stretching and lateral flexion, with the model further tested using the Hueter-Volkmann principle.

**Result:** On comparing the measurements across three different load cases (forward bending, after stretching and lateral flexion) in patients with AS after thoracic lumbar protrusion deformity and the original X-ray images, no difference was found between the model of deformation and real patient movement displacement across the vertebral body. On simulating the stress distribution, the measured T10-L4 vertebral body stress values at every point in the injured vertebrae were, on the whole, directed at both the upper and lower ends and exhibited a decreasing trend, and the stress distribution gradually decreased from the injured vertebrae (T12 and L1) to the upper and lower ends.

**Conclusion:** The accuracy of the research model is high, the geometric similarity is good and relevant applied anatomy can be undertaken using the model parameter measurement. This study provides a successful example of the application of digital technology in the field of spinal deformity and a novel idea for the treatment of AS-related kyphosis.

## 1 Introduction

Ankylosing spondylitis (AS) is a chronic inflammatory disease that primarily affects the spine and can lead to thoracolumbar kyphosis deformity in later stages (Södergren et al., 2021; Tang et al., 2022). The condition typically presents with symptoms of sacroiliac joint pain that gradually progresses upwards, involving the lumbar, thoracolumbar, thoracic and cervical regions of the spine (Chang et al., 2005; Qian et al., 2012; Liu et al., 2021). The mainstay of treatment for late-stage AS involves surgery or orthosis. Osteotomy is a common surgical method used to treat advanced thoracolumbar kyphosis in patients with AS (Liu et al., 2020; Zhang et al., 2020; Li et al., 2022). Combined vertebral arch and vertebral body osteotomy represents a recent advancement in late orthopaedic surgery for thoracic and lumbar kyphosis related to AS (Bourghli et al., 2021). Thoracolumbar kyphosis deformity is a challenging condition to manage; however, surgical intervention can provide significant relief in patients with advanced disease.

Surgery is required to not only correct kyphosis but also maintain sagittal balance (Hiyama et al., 2019). Osteotomy angles that are either too large or too small have the potential to affect spine biomechanics following surgery; however, there exist few relevant studies on the specific effects. With the development of informative technologies, computer simulation modelling and biomechanical analysis, their effective integration within medicine has had a tremendous impact on spinal surgery. Using the finite element method of biomechanics, it is possible to perform simulations for specific cases, including in terms of orthopaedic devices and strategies to predict postoperative orthopaedic outcomes, analysing the influence of intraoperative parameter selection on outcomes and guiding surgical planning.

This study utilises three-dimensional (3D) digital modelling technology to conduct personalised surgical design for a patient with postoperative kyphosis deformity related to AS. Finite element analysis was employed to predict the surgical outcomes. The study presents a precise and individualised approach for surgical planning in AS-related postoperative kyphosis deformity.

## 2 Materials and experimental methods

### 2.1 Case study patient information

The patient, a 56-year-old man, was admitted to hospital mainly due to experiencing thoracolumbar kyphosis deformity for 10 years and aggravation for more than 1 year. The physical examination indicated no obvious scoliosis in the lumbar segment, and the spine presented with a round protuberous kyphosis deformity, positive for spinous process and paravertebral tenderness in thoracolumbar 1, and negative for pain in both lower limbs. The muscle strength of the limbs was normal, the reflexes of the knee tendon and Achilles tendon were weakened and the residual sensation and reflex were normal. The Cobb angle was approximately 56°, and the four-word test returned positive results.

### 2.2 Equipment and related software

The equipment included a computed tomography (CT) machine (Lightspeed VCT, General Electric Company, United States) and its own measurement software (US VMT-XT-64), with the scanning parameters set as follows: layer thickness = 0.625 mm, layer distance = 0.625 mm, scanning time = 0.4 s, bulb voltage = 120 kV, current = 200 mA, matrix size = 512 × 512, imaging field = 15 × 15 cm and pixel size = 0.625 × 0.625. The acquired image data were stored on a 3T (Toshiba Canvio 3.5, Tokyo, Japan) mobile hard disk provided by the Thoracic, Lumbar and Spinal Surgery Department of the Second Affiliated Hospital of Inner Mongolia Medical University and the School of Mechanical Engineering of Dalian University of Technology. An Acer desktop was also used (CPU, AMDFX [TM]six-core, 3.5G, 8G memory, 2G video memory, Windows 7 64-bit system) along with the following 3D reconstruction software: an interactive medical image control system (MIMICS16.0, Materialise, Leuven, Belgium) and 3-matic 8.0 software (Materialise). The reverse engineering software included Geomagic Studio (V9 Demon Software Co., LTD. Rock Hill, South Carolina, United States) and Pro-E4.0 (PTC, Needham, Massachusetts, United States), and the finite element analysis software used was ANSYS14.0.

### 2.3 Modelling procedure

#### 2.3.1 Three-dimensional reconstruction of the spine

**FIGURE 1:**
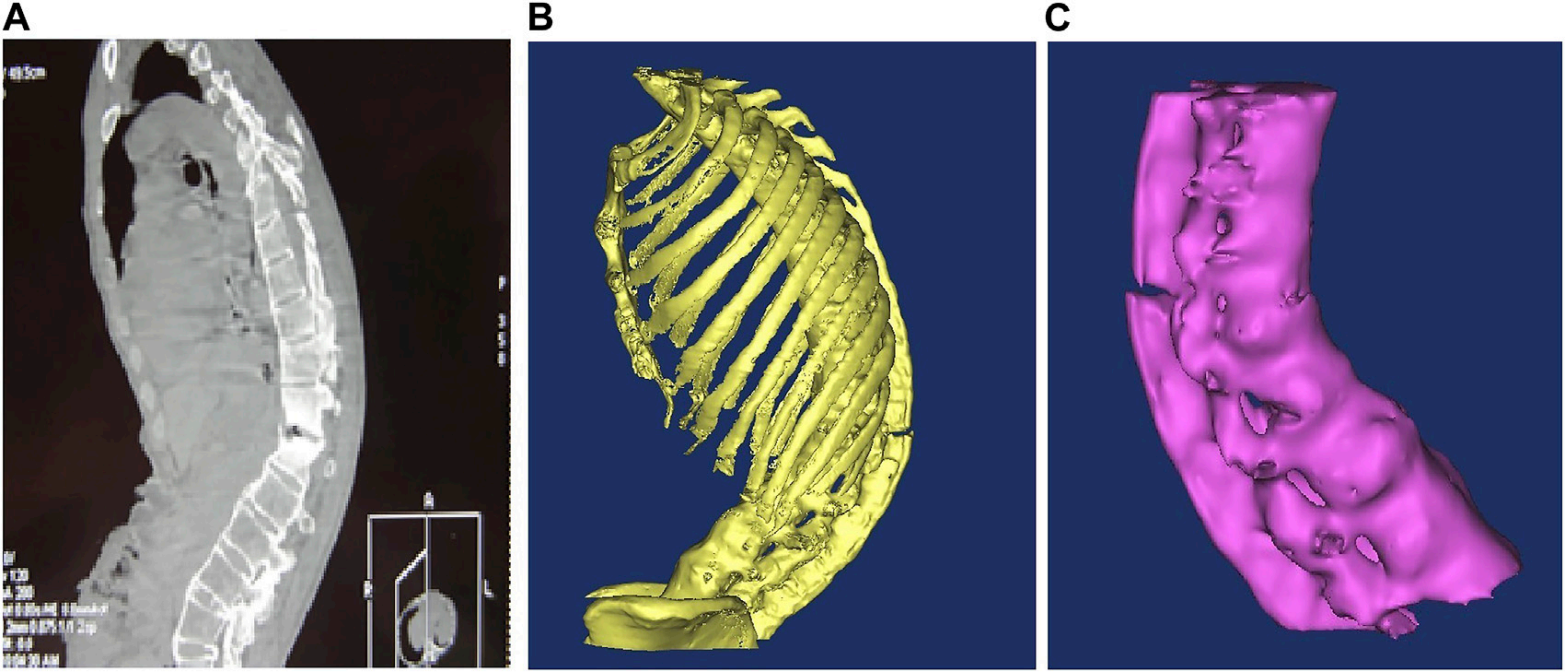
**(A)** Ankylosing spondylitis CT scan lateral radiographs; **(B)** 3D model after noise removal and boundary smoothing; **(C)** bone independent boundary modification was removed and T10-L4 segments were retained.

In the multi-slice CT workstation, the contrast was adjusted to ensure that the measurement structure was clear and developed before the file was saved in DICOM format. The saved file was then imported into the MIMICS16.0 3D reconstruction software and different masks were assigned to different structures using the threshold setting. The boundaries of the different masks were manually modified through the coronal plane, sagittal plane and 3D graphics, and the final reconstruction was performed after modification. Three different sections (horizontal plane, coronal plane and sagittal plane) in the software were used for image registration, measurement points were determined, the tool toolbar was activated and related diameter and angle indices were measured. The complete 3D kyphosis model building process is shown in Figure 1.

#### 2.3.2 Surgical simulation

**FIGURE 2:**
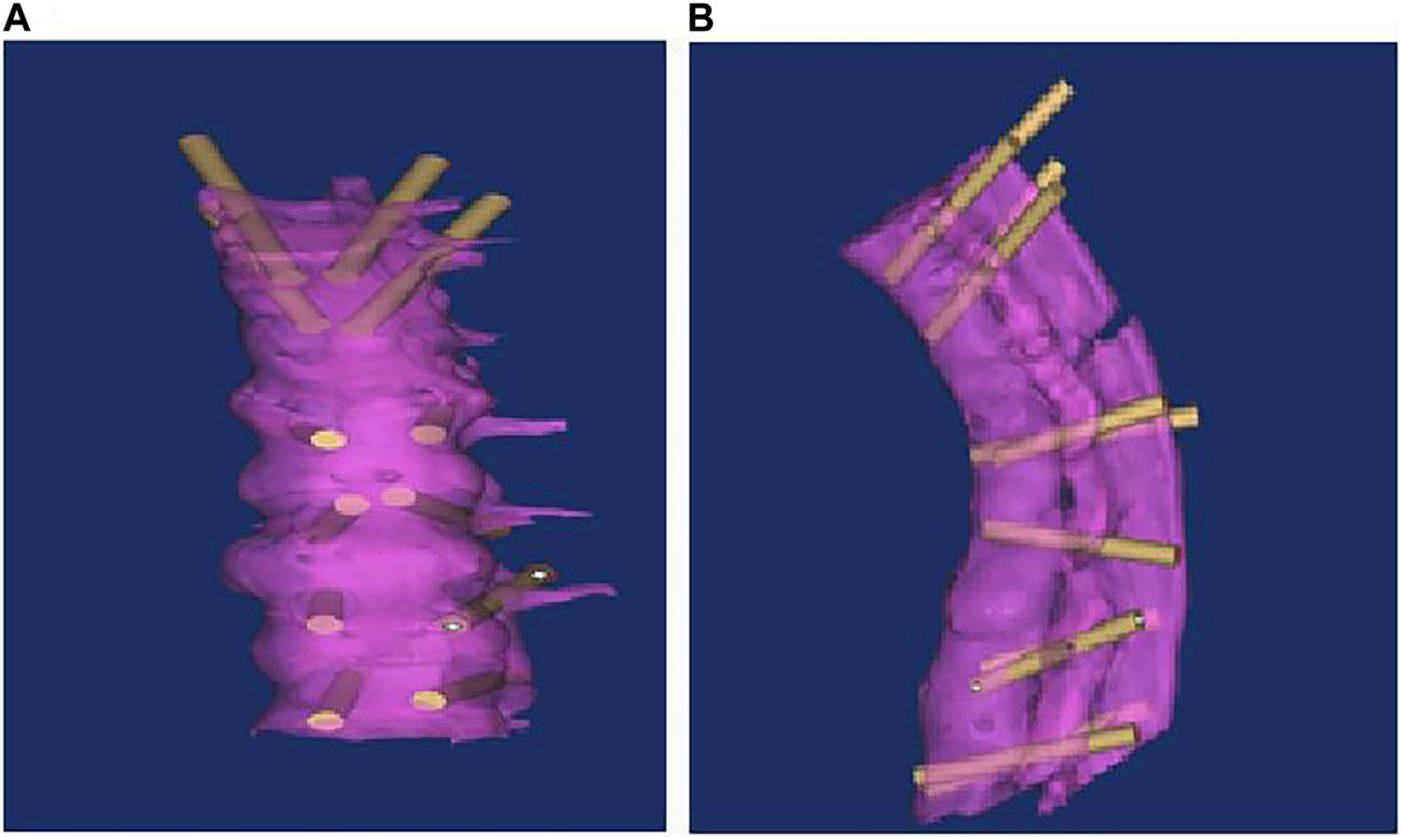
Anteriorly after screw registration [coronal plane **(A)** and sagittal plane **(B)**].

Osteotomy and pedicle screw positioning were performed on the lower margin lamina of thoracic 12 (T12), one disc of T12/lumbar 1 (L1) and the upper 1/2 of the L1 vertebral body and pedicle. The completed 3D imaging was imported from the MIMICS software into the 3-matic software. In the MIMICS software, computer-assisted design tools were used to design the screw model, and the 3D stereoscopic model was processed transparently, with 3D stereoscopic images, in the horizontal, coronal and sagittal plane, used for 3D registration. In strict accordance with the principle of clinical surgical nail placement, the screw did not penetrate the vertebral canal through the pedicle and the front of the screw did not penetrate the vertebral cortex. The position of the designed pedicle screw was adjusted to simulate the screw entry point and calculate the screw implantation angle (horizontal angle and sagittal angle) and screw implantation length (Figure 2).

### 2.4 Surgical procedure

Following successful endotracheal intubation under general anaesthesia, the patient was placed in the prone position. The abdomen and chest were padded with cotton sticks, and the upper limbs were flexed and fixed on the abduction frame. The skin in the conventional operative field was disinfected with 2% iodophor three times, the surgical sheet was spread with a surgical towel and the protective film was affixed. An incision with a length of approximately 20 cm was made at the posterior median of T10-L4 spinous processes in the chest. The skin was cut open, the subcutaneous tissue was electrically cut, the supraspinous ligament was cut at the midline between the thoracolumbar fascia and spinous process, and subperiosteal dissection was performed along the side of the spinous process and lamina using a periosteal stripper. Both sides of the vertebral lamina and the superior and inferior articular processes were exposed. During the operation, the size and progress angle of the internal fixation screw designed according to the preoperative data were inserted into the right vertebral pedicle of T10, T11, T12, L2, L3, and L4. The intraoperative radiographs confirmed that the fixed vertebral body and the positioning direction were correct. The positioning needle was removed, drilling holes were made along the designed entry point and direction and taps were created. The four walls and bottom of the pedicle probe were made of bone. A universal screw (6.0 × 40 mm) was inserted into the left pedicle of T10, and 6.0 × 45-mm screws were inserted into the left pedicle of the 11th and 12th thoracic vertebrae. A 6.5 × 50-mm universal pedicle screw was then inserted into the left pedicle of the L2, L3 and L4 vertebrae, and the same screw was inserted on the right side of the same vertebra in the same manner. During the operation, both sides of the L1 vertebra, the lower margin of the T12 vertebra and both sides of the T12-L1 intervertebral disc were carefully separated. According to the “triangle” bone block removed by preoperative design, the lamina at the lower margin of T12, the disc at T12-L1, the upper half of the L1 vertebral body and vertebral pedicle, the corresponding lamina and the yellow ligament were removed, the upper and lower vertebral canals at the osteotomy were expanded, the pre-curved orthopaedic rod was placed on both sides, part of the nuts were locked, and the osteotomy space was closed by pressure (after the osteotomy face is closed, the residual spinous process, lamina and vertebral body osteotomy surface can come into close contact; spinal canal continuity remains intact) and the lock fixed. The intraoperative radiography results confirmed that the position and fixation of the 12 screws were good, the osteotomy and orthosis were good and the physiological curvature of the lumbar spine was restored. The laminae on both sides of the T11-L3 vertebrae were chiselled into a irregular skeletal surface using a bone knife. Prefabricated bone strips and allogeneic bones were laid on the surface and transverse connecting rods were installed.

### 2.5 Establishment of the three-dimensional finite element model

The 3D model of the thoracolumbar segment in patients with AS was established using the MIMICS16.0 software, with the 3D automatic registration function of the software used for automatic registration. After registration, the 3-matic 8.0 software was opened directly, the surface mesh was divided into vertebrae, intervertebral discs and endoplants, and the surface mesh was optimised using the auto-remesh function. Following surface mesh division, volume mesh division was performed before each structure was imported into the MIMICS16.0 software. The grayscale assignment function of the software was used to assign material values to the vertebrae and intervertebral discs, and the existing ligament material parameters were inputted (Table 1).

**TABLE 1:** Material parameter.

Site	Element type	Young’s modulus (Mpa)	Poisson’s ratio (μ)
Cortical bones	8-node solid	12,000	0.30
Cancellous bones	8-node solid	345	0.20
End plate	8-node solid	12,000	0.30
ALL	2-node	20	0.30
PLL	nonlinear	70	0.30
ISL	cable elements	28	0.30
SSL	cable elements	28	0.30
LIL	cable elements	28	0.30
RIL	cable elements	28	0.30

ALL, anterior longitudinal ligament; PLL, posterior longitudinal ligament; ISL, Iliolumbar ligament; SSL, supraspinous ligament; LIL, left iliolumbar ligament; RIL, right iliolumbar ligament.

### 2.6 Finite element model verification

Because the finite element model was established when the patient was lying flat, the patient was asked to adopt the same position during model verification to eliminate any change in spine shape caused by body weight. After CT was performed, the patient lay flat on the bed, underwent positive and lateral X-rays in a supine position and positive and lateral X-rays while flexed laterally, and the pelvis was fixed according to routine procedures. The patient was photographed in the maximum lateral flexure position as far as possible. The patient gave their informed consent for all experimental procedures, which were also approved by the ethics committee of the school.

The established finite element model was simulated and loaded, and the lower-edge surface of the L4 vertebra was fixed. A 500-N load was continuously applied to the upper surface of the T10 vertebra and was evenly distributed to each node on the surface. While the 500-N load was vertically loaded, a 15-Nm torque was applied to the upper surface of the T10 vertebra and the load was divided into three conditions: forward bending, backward extension and lateral bending.

Qualitative and quantitative methods were used to represent displacement, which was measured using a deformation distribution nephogram.

The deviation distance between the body and heart of each T10-L4 vertebra and the perpendicular line of the sacrum was measured to compare the differences between the model and radiographs.

### 2.7 Stress analysis of kyphotic deformity in ankylosing spondylitis

A finite element model was established to simulate loading, and the lower-edge surface of the L4 vertebra was fixed. A 500-N load was continuously applied to the upper surface of the T10 vertebra and was evenly distributed to each node on the surface. The vertical stress loading of the normal human body was simulated while the 500-N load was vertically loaded. Qualitative and quantitative methods were used to represent the displacement, which was represented and measured using a stress distribution nephogram.

### 2.8 Statistical method

The distances between the body and heart of each vertebra measured by the finite element model and the results measured using X-rays indicated that all the measured data obeyed a normal distribution. Variance analysis was used to determine whether there were any differences between the two groups, and the significance level was set at *p* > 0.05.

## 3 Results

### 3.1 Anatomical and surgical design parameters

Details of the clinical application of anatomical parameters in the AS-related kyphotic deformity of T10–L4 are presented in Table 2, and details of the measurement of the pre-surgery design parameters for the kyphosis of T10–L4 in AS are presented in Table 3.

**TABLE 2:** Clinical application of ananatomical parameters in kyphotic deformity of T10-L4 ankylosing spondylitis.

Vertebral body	Nail setting angle (unit: °)	Nail diameter (unit: mm)	Nail length (unit: mm)
T10	L12.42R6.11	L6.69R6.55	L53.25R48.84
T11	L13.86R5.62	L10.10R8.64	L51.13R45.87
T12	L7.82R7.04	L8.55R8.97	L53.69R47.90
L1	L3.90–4.98	L8.16–9.60	L68.52–68.90
	R7.23–9.40	R8.83–8.35	R58.51–68.69
L2	L10.39R10.77	L10.42R9.68	L80.13R80.23
L3	L9.11R16.8	L10.59R12.02	L83.52R69.07
L4	L6.45R14.74	L11.90R14.15	L81.71R76.81

**TABLE 3:** Measurement of surgical design parameters before surgery for kyphosis of T10-L4 ankylosing spondylitis.

Vertebral body	Left side (unit: mm)	Right side (unit: mm)
T10	diameter: 6.0 length: 40	diameter: 6.0 length: 40
T11	diameter: 6.0 length: 45	diameter: 6.0 length: 45
T12	diameter: 6.0 length: 45	diameter: 6.0 length: 45
L2	diameter: 6.5 length: 50	diameter: 6.5 length: 50
L3	diameter: 6.5 length: 50	diameter: 6.5 length: 50
L4	diameter: 6.5 length: 50	diameter: 6.5 length: 50

All internal fixation screws were universal pedicle screws.

### 3.2 Surgical evaluation of kyphotic deformity of T10-L4 in ankylosing spondylitis

The operation time was 7 h 00 min, and the blood loss was approximately 500 ml. The patient was generally in good condition with no postoperative symptoms of cerebrospinal fluid leakage, no numbness in the lower limbs, good movement of the toes on both feet and no complaints of discomfort. The postoperative radiographs indicated good internal screw fixation, a preoperative Cobb angle of 56°, a postoperative Cobb angle of 20° and good postoperative correction.

### 3.3 Establishment of finite element model for kyphotic deformity of T10-L4 in ankylosing spondylitis

**FIGURE 3:**
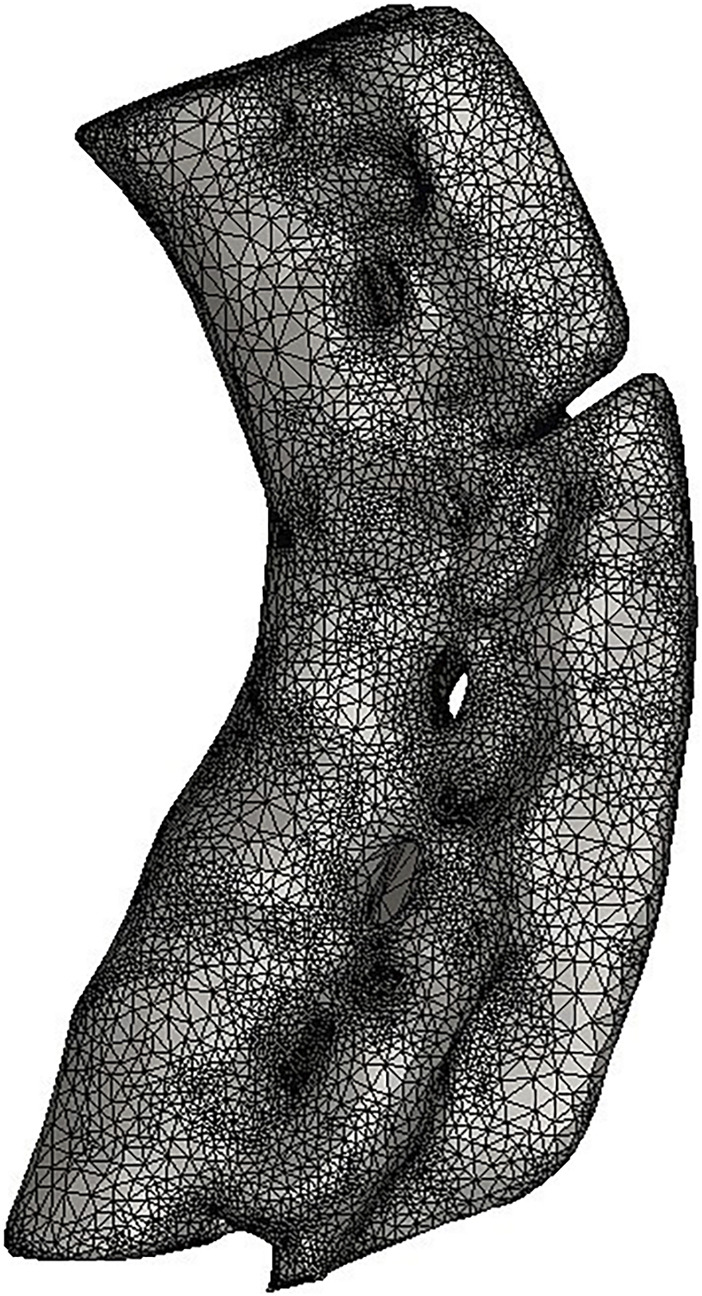
Establishment of 3D finite element model.

The finite element model included seven vertebral bodies (T10-L4) and six intervertebral discs. The anterior longitudinal ligament, posterior longitudinal ligament, supraspinous ligament, interspinous ligament and ligamentum flavum. The vertebrae and intervertebral discs were meshed, and 32,500 solid units were generated. The results for the finite element model are shown in Figure 3.

### 3.4 Validation of the finite element model for kyphotic deformity of T10-L4 in ankylosing spondylitis

**FIGURE 4:**
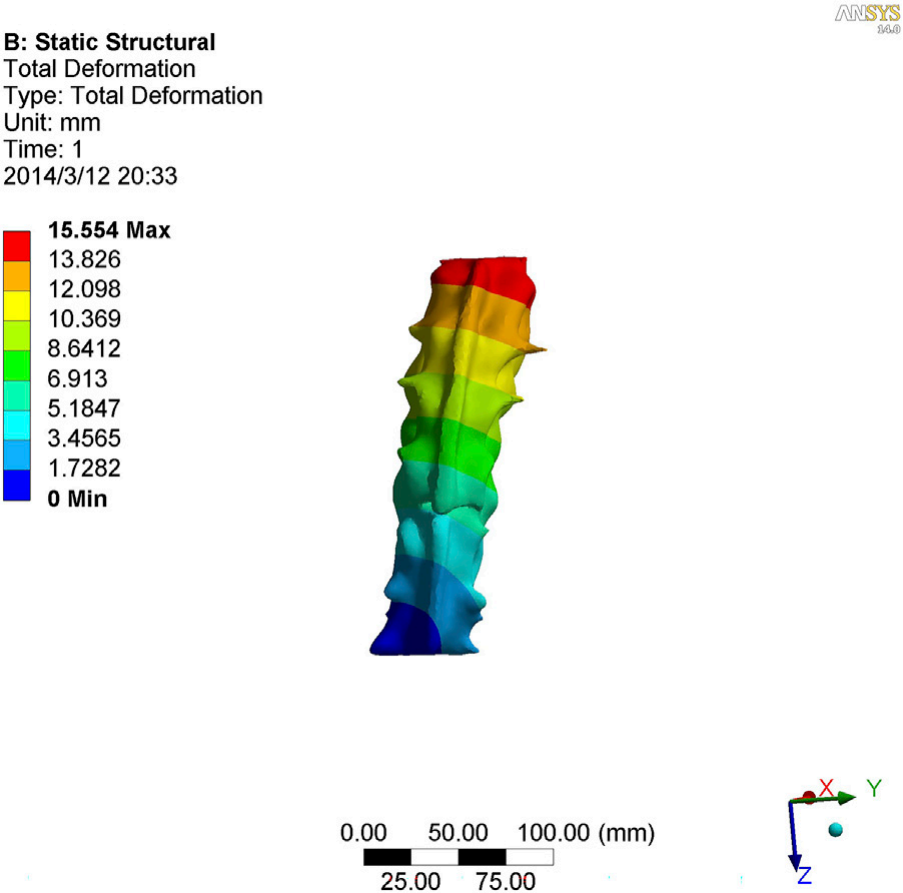
Nephogram of lateral curvature in kyphotic deformity of T10-L4 ankylosing spondylitis.

**FIGURE 5:**
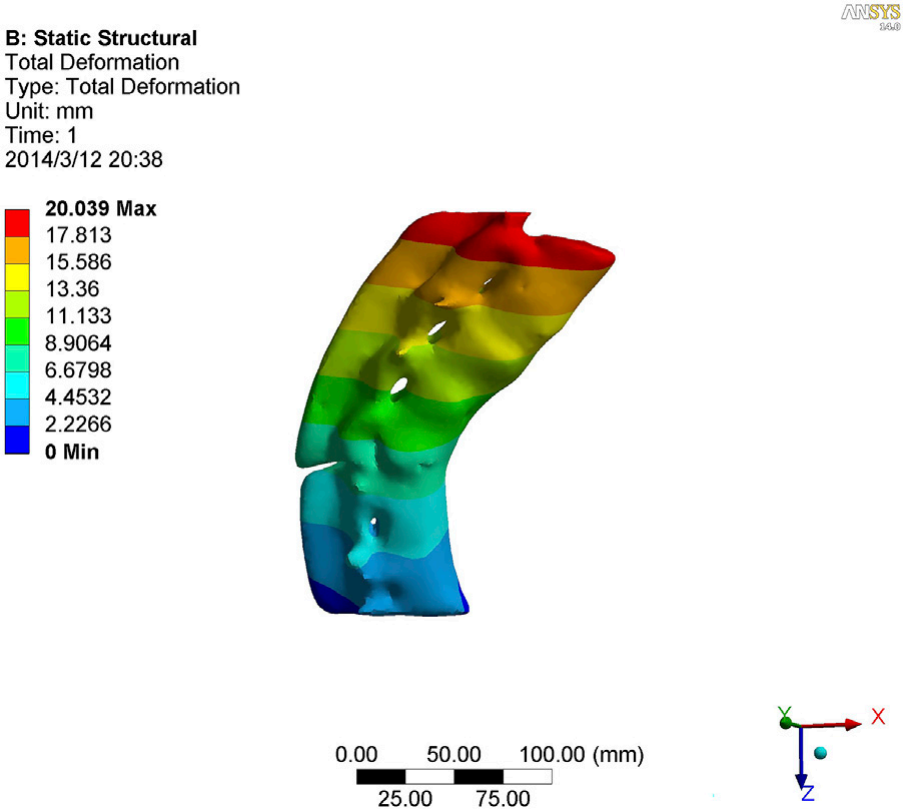
Nephogram of anterior flexion in kyphotic deformity of T10-L4 ankylosing spondylitis.

**FIGURE 6:**
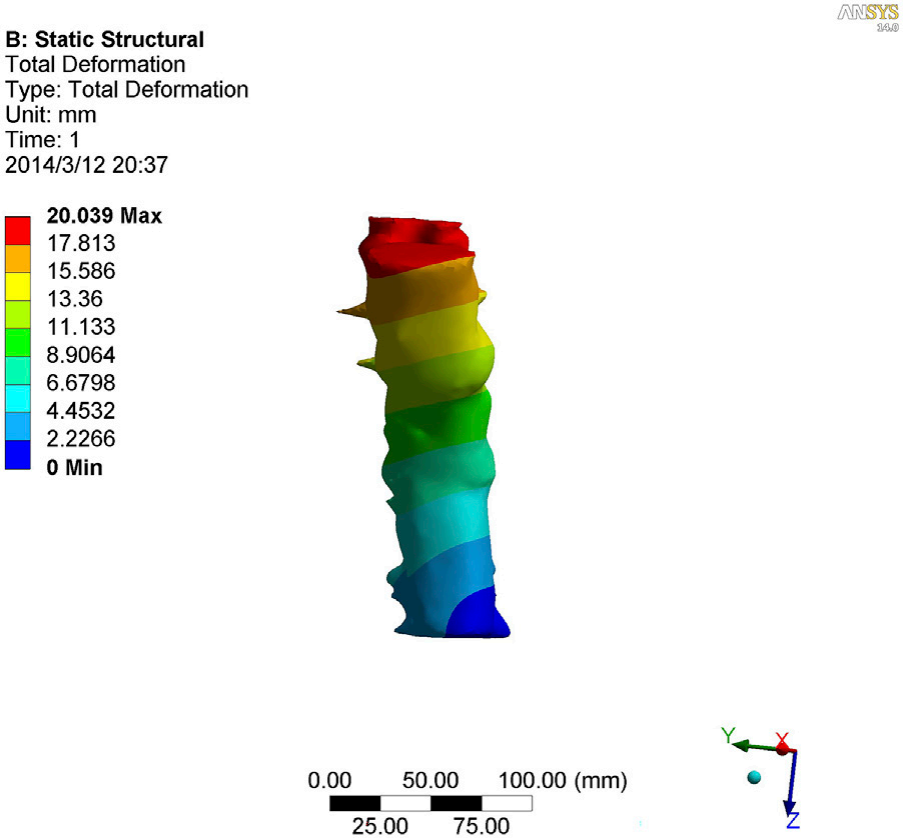
Nephogram of posterior extension of kyphotic deformity of T10-L4 ankylosing spondylitis.

Table 4 shows the migration distance between the body mass and the migration of each vertebra and vertical line of the sacrum. A paired *t*-test was used with a level set at 0.05, which determined that there was no statistical difference between the two groups of data. Three movements were compared between the two groups, and the shift distance of the body and heart of each T10-L4 vertebra relative to the vertical line of the sacrum was measured. There was no difference between the comparison model and the distance measured using X-ray. This model was consistent with a real patient model (Figures 4–6).

**TABLE 4:** Comparison between model and radiographs of vertebral body body and heart deviation relative to sacral midvertical distance (cm).

	Forward flexion position		Lateral flexion		Back extension	
	X-ray	Model	X-ray	Model	X-ray	Model
T10	1.2	1.0	5.2	5.1	−1.2	−1.0
T11	2.4	2.3	6.7	6.2	0.5	0.6
T12	3.6	3.5	7.1	7.4	1.2	1.4
L1	3.9	4.0	8.5	8.4	2.5	2.6
L2	4.2	4.5	7.1	6.9	4.1	4.5
L3	3.9	3.8	8.2	8.6	4.7	4.9
L4	3.2	3.1	7.1	7.5	5.1	5.3
	t = 1.324; *p* = 0.235		t = 1.564; *p* = 0.124		t = 0.954; *p* = 0.684	

### 3.5 Stress analysis of kyphotic deformity in ankylosing spondylitis

**FIGURE 7:**
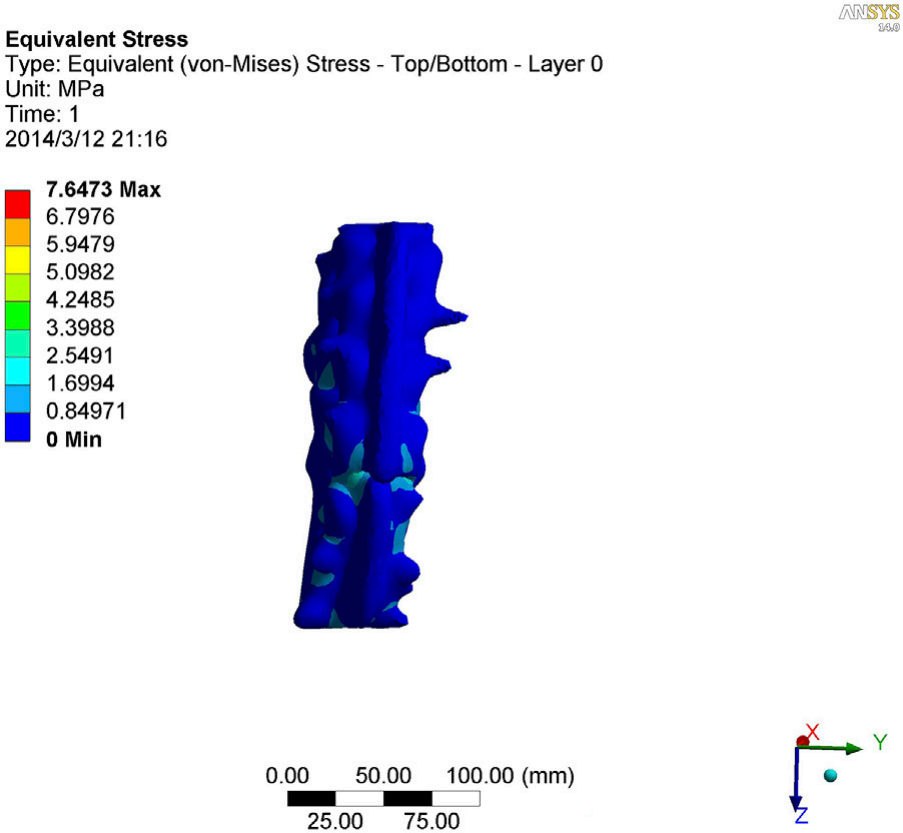
Nephogram of vertical stress distribution after loading in kyphotic deformity of T10-L4 ankylosing spondylitis.

**FIGURE 8:**
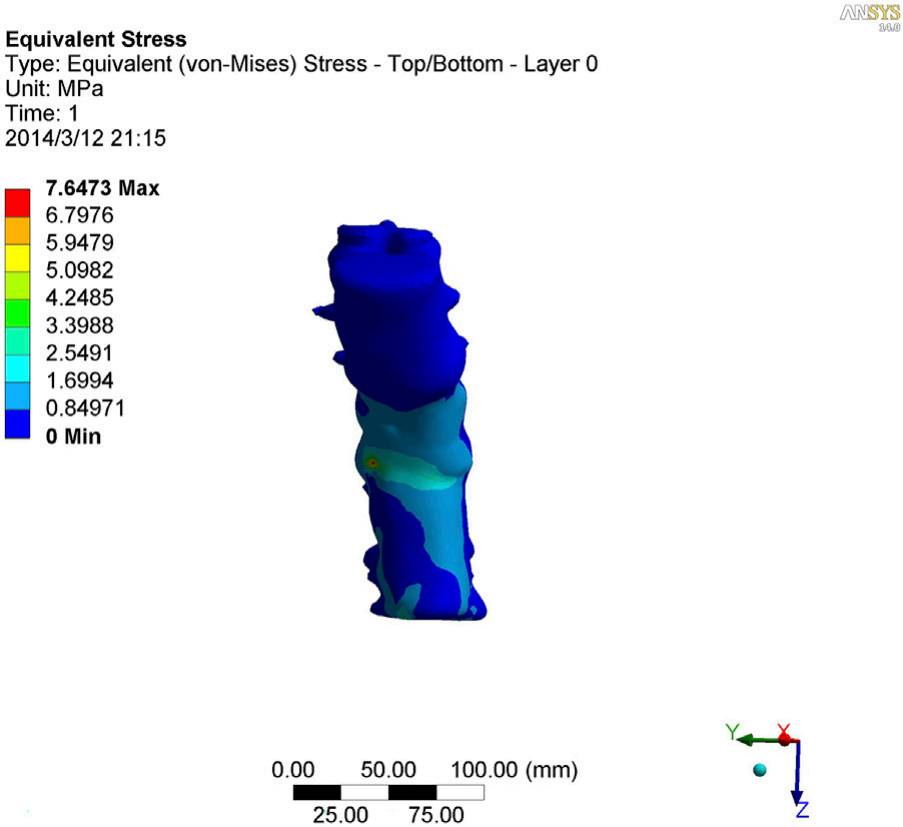
Nephogram of vertical stress loading in kyphotic deformity of T10-L4 ankylosing spondylitis.

**FIGURE 9:**
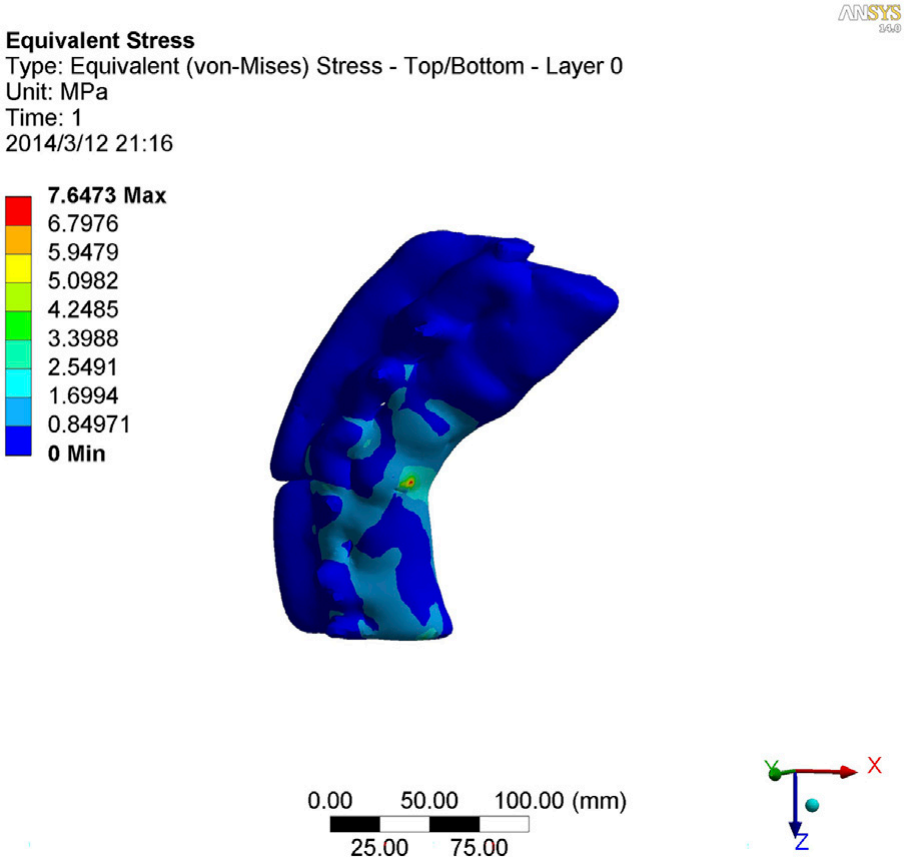
Lateral view of vertical stress loading in kyphotic deformity of T10-L4 ankylosing spondylitis.

As shown in Figures 7–9, the stress on the anterior edge of the T12-L1 vertebrae was the highest, with a maximum point stress of 7.643 MPa, followed by that on the L2-L3 vertebrae (2.58 MPa). In the entire study segment, the stress distribution gradually decreased from the injured vertebrae (T12 and L1) to the upper and lower ends. For T10-L4, the spinous process stress was relatively dispersed, with a maximum stress point of 0.032 MPa, whereas the L3 and L4 pedicle stress relative to the spinous process was concentrated, with a maximum stress value of 1.98 MPa, and the lower margin of L4 vertebral body stress was equivalent to that of the pedicle stress, with a maximum value of 1.78 MPa.

## 4 Discussion

Three-dimensional digital models can be used to simulate a variety of surgical procedures, provide a quantitative analysis of the results of simulated surgery and allow repeated manipulation of the model without fear of model damage. In this study, a 3D digital model of thoracolumbar kyphosis deformity related to AS was selected. The internal fixator of the nail rod was implanted into the model, and mesh division and finite element analysis were performed.

The establishment of a finite element model ensures consistency with the real object in terms of both appearance and mechanical properties. Effective verification following the establishment of the finite element model is crucial for subsequent biomechanical research (Varga et al., 2015; Wang et al., 2015; Wang et al., 2018). The appearance of the T10-L4 finite element model established in this study was superimposed on the thin-slice CT image data of real patients processed using medical image processing software, meaning the appearance was consistent with the spinal model of real patients. To further verify the consistency between the model and the actual patient’s mechanical properties, the coronal plane line morphology of the body and heart of each vertebra on the preoperative clinical lateral flexion radiographs of patients with AS-related kyphosis and the finite element simulation lateral flexion pictures were compared and the migration distance of the body and heart of each vertebra relative to the vertical line of the sacrum was calculated. There was no difference between the comparison model and the distance measured using X-ray. The model was consistent with a real patient model, and the mechanical properties of the model presented herein are consistent with those of a real model (Hambli, 2014; Podshivalov et al., 2014).

To further study the inherent biomechanical characteristics of AS-related kyphosis in the T10-L4 vertebrae, the model loading was simulated, with the lower edge surface of the L4 vertebra fixed, a 500-N load was continuously applied on the upper surface of the T10 vertebra and the load was evenly distributed on each node of the surface. At the same time, when the 500-N load was vertically loaded, as revealed in the stress distribution histogram, the stress at the anterior edge of the T12-L1 vertebrae was the highest, and the maximum point stress value was 7.643 MPa. The main reason for this was that the compression fracture at the anterior edge of the T12-L1 vertebrae, spinal deformity, common stress on the normal articular process, and upper and lower endplates of the vertebrae are concentrated at the anterior edge of the vertebrae. Therefore, compressive bone vertebrae have the most concentrated stress at the anterior edge of the body, which is also the most prone to further injury, leading to spinal instability. The second reason relates to the anterior edge of the L2-L3 vertebrae, the maximum stress value of which is 2.58 MPa, indicating that with the fracture of the T12-L1 vertebrae, stress is borne downwards along the anterior edge of the L2-L3 vertebrae, which also becomes the relative concentration point of stress, thus aggravating the injury. Therefore, the entire spinal segment, except T12-L1, is easily compressed again, and the lower L2-L3 vertebrae are prone to secondary compression fractures. In the entire study segment, the stress distribution gradually decreased from the injured vertebrae (T12 and L1) to the upper and lower ends.

For T10-L4, the spinous process stress is relatively dispersed, and the spinous process is less prone to injury or fracture. The stress in the L3 and L4 pedicles was more concentrated than that in the spinous process, with a maximum value of 1.98 MPa, indicating that this pedicle segment is also prone to fracture injury.

This study utilised 3D digital technology and finite element analysis to establish individualised spinal models for patients with AS, design precise osteotomy correction plans and predict and evaluate surgical outcomes. This provides a new approach for computer-assisted precision surgical treatment of postoperative kyphosis in AS.

However, as an initial exploratory study, the sample size was limited to only one case, resulting in limited clinical representation of the results. In addition, the study relied solely on computer simulations without clinical validation, and certain assumptions and simplifications were made in both the model and calculations. Moreover, the surgical risks and long-term effects were not comprehensively assessed. Further research with a larger sample size is needed to validate the findings and optimise the model to bridge the gap between the potential of this technology and its clinical implementation.

## 5 Conclusion

The model established in this study realises accurate osteotomy and orthopaedic scheme design and surgical effect prediction based on computer technology. This presents a novel solution to the problem of individual differences in the treatment of AS kyphosis. This study demonstrates that it is feasible to apply digital technology to realise individualised treatments for patients.

## Data Availability

The original contributions presented in the study are included in the article/supplementary material, further inquiries can be directed to the corresponding author.
